# Detection of Illegally Manufactured Fentanyls and Carfentanil in Drug Overdose Deaths — United States, 2021–2024

**DOI:** 10.15585/mmwr.mm7348a2

**Published:** 2024-12-05

**Authors:** Lauren J. Tanz, Andrea Stewart, R. Matt Gladden, Jean Y. Ko, Lauren Owens, Julie O’Donnell

**Affiliations:** 1 Division of Overdose Prevention, National Center for Injury Prevention and Control, CDC.

**Summary**

**What is already known about this topic?**

Approximately 70% of U.S. overdose deaths in 2023 were estimated to involve illegally manufactured fentanyls (IMFs). Local reports indicate reemergence of carfentanil, a fentanyl analog.

**What is added by this report?**

Overdose deaths overall and with IMFs detected began declining in 2023. Percentages of overdose deaths with IMFs detected were stable (approximately 70%–80%) during 2021–2024, except in the West where the percentage increased from 48.5% to 66.5%. Although rare, deaths with carfentanil detected increased approximately sevenfold, from 29 during January–June 2023 to 238 during January–June 2024; 37 states reported carfentanil detection.

**What are the implications for public health practice?**

Overdose prevention efforts that address widespread presence of IMFs, including carfentanil, and can rapidly adapt to other potent opioids in the drug supply, might result in lasting reductions in overdose deaths across the United States.

## Abstract

During 2023, approximately 72,000, or nearly seven in 10, drug overdose deaths in the United States were estimated to involve illegally manufactured fentanyls (IMFs). Carfentanil, a fentanyl analog 100 times more potent than fentanyl, has reemerged in the U.S. drug supply. Using CDC’s State Unintentional Drug Overdose Reporting System data, this report describes trends in overdose deaths during January 2021–June 2024, overall and with IMFs detected, by U.S. Census Bureau region, and in deaths with carfentanil detected, in 45 states and the District of Columbia (DC). Numbers of deaths with carfentanil detected by state during January 2023–June 2024 in 49 states and DC are also reported. The number of overdose deaths with IMFs detected declined from 2022 to 2023 in the Northeast (3.2% decline), Midwest (7.8%), and South (2.8%) regions; deaths in the West increased 33.9%. The percentage of deaths with IMFs detected was steady at approximately 70%–80% in the Northeast, Midwest, and South. In contrast, the percentage of deaths with IMFs detected in the West increased from 48.5% during January–March 2021 to 66.5% during April–June 2024. Overdose deaths with carfentanil detected increased approximately sevenfold, from 29 during January–June 2023 to 238 during January–June 2024; during January 2023–June 2024, overdose deaths with carfentanil detected were reported in 37 states. Overdose prevention efforts that address the widespread presence of IMFs, including carfentanil, and can rapidly adapt to other potent opioids in the drug supply might result in lasting reductions in overdose deaths across the entire United States.

## Introduction

In 2013, illegally manufactured fentanyl and fentanyl analogs (IMFs) entered the U.S. illegal drug supply as adulterants of or replacements for white powder heroin in the Northeast and have now replaced heroin as the dominant opioid in the United States (*1*). Introduction of IMFs led to a sharp rise in overdose deaths, likely because of their high potency and rapid onset of effects (*2*,*3*). In 2023, approximately 72,000 drug overdose deaths, or nearly seven in 10, were estimated to involve fentanyl, which is primarily illegally manufactured.*^,^^†^ Provisional data project a decrease in overdose deaths in 2023 compared with the number in 2022, the first decline since 2018; the decrease appears to have continued into 2024.^§^ However, recent reemergence of carfentanil,^¶^ a fentanyl analog 100 times more potent than fentanyl (*4*), which had largely disappeared after carfentanil-involved overdose death outbreaks during 2016–2017 (*5*,*6*), might threaten this progress. Data from CDC’s State Unintentional Drug Overdose Reporting System (SUDORS) were analyzed to describe recent trends in detection of IMFs and carfentanil among overdose deaths in the United States.

## Methods

### Data Source

Data on unintentional and undetermined intent drug overdose deaths obtained from death certificates, coroner and medical examiner reports, and postmortem toxicology reports were entered into SUDORS.** These data were entered by 49 states and the District of Columbia (DC) (jurisdictions) funded through the Overdose Data to Action in States cooperative agreement.^††^

### Statistical Analyses

Numbers of all overdose deaths and numbers and percentages of overdose deaths with IMFs^§§^ detected that occurred during January 2021–June 2024 were calculated overall and by quarter (Q1 = January–March, Q2 = April–June, Q3 = July–September, and Q4 = October–December) for all jurisdictions combined and for each U.S. Census Bureau region.^¶¶^ Because data for January–June 2024 are preliminary, incomplete, and have not undergone full quality control measures,*** only percentages of overdose deaths with IMFs detected are presented for this period.^†††^ Numbers of overdose deaths with carfentanil detected overall, and with carfentanil co-detected with illegally manufactured fentanyl (IMF; excluding fentanyl analogs),^§§§^ during January 2021–June 2024 were tabulated by 6-month period. All trend analyses included 46 jurisdictions (45 states and DC).^¶¶¶^ The number of overdose deaths with carfentanil detected during January 2023–June 2024 was tabulated by state among 50 jurisdictions (49 states and DC).**** Analyses were performed using SAS software (version 9.4; SAS Institute). This activity was reviewed by CDC, deemed not research, and was conducted consistent with applicable federal law and CDC policy.^††††^

## Results

### Overdose Deaths with IMFs Detected

**FIGURE 1:**
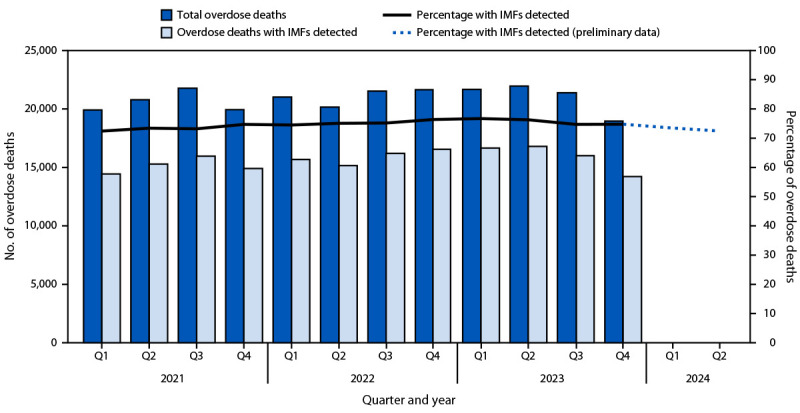
Number of drug overdose deaths overall* and number and percentage of overdose deaths with illegally manufactured fentanyls^†^ detected, by quarter of death^§^ — State Unintentional Drug Overdose Reporting System, United States,^¶^ January 2021–June 2024** **Abbreviations:** IMFs = illegally manufactured fentanyls; Q = quarter; SUDORS = State Unintentional Drug Overdose Reporting System. * A total of 251,089 overdose deaths occurred during January 2021–December 2023. Because not all overdose deaths that occurred during January–June 2024 have been entered into SUDORS, numbers of deaths overall and with IMFs detected during this time frame are underestimated and not presented. Percentages with IMFs detected during this time frame are restricted to deaths with toxicology information entered; these are expected to be accurate estimates of the percentages of overdose deaths with IMFs detected. ^†^ Fentanyl was classified as likely illegally manufactured using toxicology, scene, and witness evidence. For the 9.9% of deaths with fentanyl detected that had insufficient evidence for classification as illegal or prescription, fentanyl was classified as illegal because the majority of fentanyl overdose deaths involve illegal fentanyl. All fentanyl analogs except pharmaceutical analogs (i.e., alfentanil, remifentanil, and sufentanil) were included as IMFs. Among deaths with IMFs detected, 98.4% had IMFs listed as a cause of death. ^§^ Q1: January–March; Q2: April–June; Q3: July–September; Q4: October–December. ^¶^ Forty-six jurisdictions: Alabama, Alaska, Arizona, Arkansas, Colorado, Connecticut, Delaware, District of Columbia, Florida, Georgia, Hawaii, Idaho, Illinois, Indiana, Iowa, Kansas, Kentucky, Louisiana, Maine, Maryland, Massachusetts, Michigan, Minnesota, Mississippi, Missouri, Montana, Nebraska, Nevada, New Hampshire, New Jersey, New Mexico, New York, North Carolina, Ohio, Oklahoma, Oregon, Pennsylvania, Rhode Island, South Carolina, South Dakota, Tennessee, Utah, Vermont, Virginia, Washington, and West Virginia. For inclusion, jurisdictions were required to report ≥75% of deaths in their jurisdiction in each 6-month period during January 2021–December 2023. ** Preliminary data were downloaded from SUDORS on November 26, 2024; 2024 data in this figure were restricted to deaths with toxicology information entered and represent approximately 55% of expected overdose deaths.

During January 2021–December 2023, a total of 251,089 unintentional and undetermined intent drug overdose deaths occurred in 46 jurisdictions, including 188,082 (74.9%) with IMFs detected. Overdose deaths with IMFs detected increased 4.9%, from 60,674 in 2021 to 63,674 in 2022 and were stable from 2022 to 2023 (63,734) (Figure 1). However, during 2023, overdose deaths with IMFs detected peaked in Q2 at 16,814, declined 4.7% in Q3 to 16,019, and declined another 11.2% in Q4 to 14,229. The number of deaths with IMFs detected was 7.8% lower during the second half of 2023 (30,248) than during the second half of 2022 (32,802). Overall fatal drug overdose trends were similar to trends in deaths with IMFs detected during 2021–2023; during 2023, overdose deaths peaked in Q2 and declined thereafter.

### Overdose Deaths with IMFs Detected, by U.S. Census Bureau Region

**FIGURE 2:**
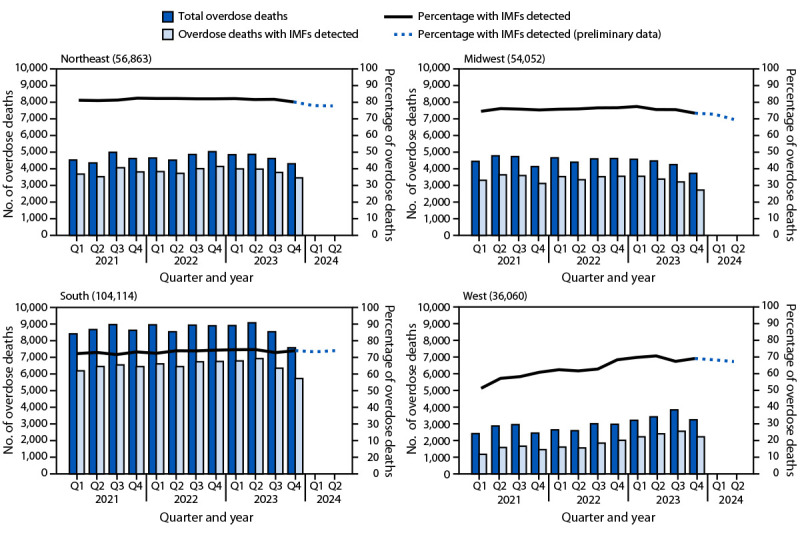
Number of drug overdose deaths overall*^,†^ and number and percentage of overdose deaths with illegally manufactured fentanyls^§^ detected, by U.S. Census Bureau region^¶^ and quarter of death** — State Unintentional Drug Overdose Reporting System, United States,^††^ January 2021–June 2024^§§^ **Abbreviations: **IMFs = illegally manufactured fentanyls; Q = quarter; SUDORS = State Unintentional Drug Overdose Reporting System. * A total of 251,089 drug overdose deaths occurred during January 2021–December 2023. Sample sizes provided in each panel represent the number of overdose deaths in that region during January 2021–December 2023. The number of deaths should not be compared across regions because population size varies by region, and not all jurisdictions in each region were included in the analysis. ^†^ Because not all overdose deaths that occurred during January–June 2024 have been entered into SUDORS, numbers of deaths overall and with IMFs detected during this time frame are underestimated and not presented. Percentages with IMFs detected during this time frame are restricted to deaths with toxicology information entered; these are expected to be accurate estimates of the percentages of overdose deaths with IMFs detected. ^§^ Fentanyl was classified as likely illegally manufactured using toxicology, scene, and witness evidence. For the 9.9% of deaths with fentanyl detected that had insufficient evidence for classification as illegal or prescription, fentanyl was classified as illegal because the majority of fentanyl overdose deaths involve illegal fentanyl. All fentanyl analogs except pharmaceutical analogs (i.e., alfentanil, remifentanil, and sufentanil) were included as IMFs. Among deaths with IMFs detected, 98.4% had IMFs listed as a cause of death. ^¶^ https://www2.census.gov/geo/pdfs/maps-data/maps/reference/us_regdiv.pdf ** Q1: January–March; Q2: April–June; Q3: July–September; Q4: October–December. ^††^ Forty-six jurisdictions were included. *Northeast Region:* Connecticut, Maine, Massachusetts, New Hampshire, New Jersey, New York, Pennsylvania, Rhode Island, and Vermont. *Midwest Region:* Illinois, Indiana, Iowa, Kansas, Michigan, Minnesota, Missouri, Nebraska, Ohio, and South Dakota. *South Region: *Alabama, Arkansas, Delaware, District of Columbia, Florida, Georgia, Kentucky, Louisiana, Maryland, Mississippi, North Carolina, Oklahoma, South Carolina, Tennessee, Virginia, and West Virginia. *West Region:* Alaska, Arizona, Colorado, Hawaii, Idaho, Montana, Nevada, New Mexico, Oregon, Utah, and Washington. For inclusion, jurisdictions were required to report ≥75% of deaths in their jurisdiction in each 6-month period during January 2021–December 2023. ^§§^ Preliminary data were downloaded from SUDORS on November 26, 2024; 2024 data in this figure were restricted to deaths with toxicology information entered and represent approximately 55% of expected overdose deaths.

**Northeast, Midwest, and South.** During January 2021–June 2024, IMFs were detected in 81.5% of overdose deaths in the Northeast, 75.4% in the Midwest, and 74.9% in the South regions; percentages of overdose deaths with IMFs detected were stable throughout the time frame (Figure 2). From 2021 to 2022, the number of deaths with IMFs detected increased 4.1% in the Northeast (from 15,269 to 15,900), 2.1% in the Midwest (from 13,825 to 14,119), and 3.6% in the South (from 25,631 to 26,543). Subsequently, from 2022 to 2023, the number of deaths with IMFs detected decreased 3.2% in the Northeast (to 15,397), 7.8% in the Midwest (to 13,022), and 2.8% in the South (to 25,789). Declines were sharpest in the second half of 2023: compared with the second half of 2022, deaths with IMFs detected decreased 11.2% in the Northeast (8,245 to 7,323), 16.1% in the Midwest (7,160 to 6,008), and 10.5% in the South (13,492 to 12,077).

**West.** In the West Region, the percentage of overdose deaths with IMFs detected increased from 48.5% during Q1 2021 to 66.5% during Q2 2024. The number of overdose deaths with IMFs detected increased 19.5% from 2021 (5,949) to 2022 (7,112) and increased 33.9% from 2022 to 2023 (9,526), representing a 60.1% increase from 2021 to 2023. However, the number of deaths with IMFs detected decreased 13.0% in late 2023, from 2,588 in Q3 to 2,252 in Q4.

### Overdose Deaths with Carfentanil Detected

**FIGURE 3:**
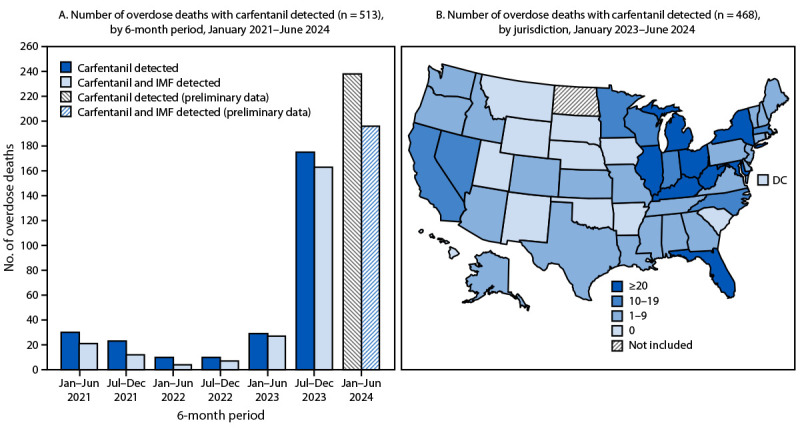
Number of drug overdose deaths with carfentanil detected,* by 6-month period of death (A)^†,§^ and jurisdiction (B)^¶^ — State Unintentional Drug Overdose Reporting System, United States, January 2021–June 2024**^,††^ **Abbreviations:** DC = District of Columbia; IMF = illegally manufactured fentanyl; SUDORS = State Unintentional Drug Overdose Reporting System. * IMF includes illegally manufactured fentanyl but does not include illegally manufactured fentanyl analogs. ^†^ For inclusion, jurisdictions were required to report ≥75% of overdose deaths in their jurisdiction in each 6-month period from January 2021 through December 2023. The percentage of overdose deaths reported for January–June 2024 was not calculated because data are preliminary and incomplete. ^§^ Forty-six jurisdictions: Alabama, Alaska, Arizona, Arkansas, Colorado, Connecticut, Delaware, District of Columbia, Florida, Georgia, Hawaii, Idaho, Illinois, Indiana, Iowa, Kansas, Kentucky, Louisiana, Maine, Maryland, Massachusetts, Michigan, Minnesota, Mississippi, Missouri, Montana, Nebraska, Nevada, New Hampshire, New Jersey, New Mexico, New York, North Carolina, Ohio, Oklahoma, Oregon, Pennsylvania, Rhode Island, South Carolina, South Dakota, Tennessee, Utah, Vermont, Virginia, Washington, and West Virginia. ^¶^ Jurisdictions were included if they reported any drug overdose deaths to SUDORS during January 2023–June 2024. North Dakota was not included because it is not funded for SUDORS. For January–December 2023, all jurisdictions except one reported ≥90% of drug overdose deaths in their jurisdiction. For January–June 2024, the percentage of overdose deaths reported by jurisdiction was not calculated because data are preliminary and incomplete. Texas and Wyoming only include data on deaths during January–June 2024 per funding agreement. ** Data on drug overdose deaths that occurred in 2023 are final. Carfentanil was detected in overdose deaths in 25 states in 2023, totaling 210 deaths. Two states (Florida and West Virginia) reported ≥20 deaths, seven (Illinois, Indiana, Kentucky, Maryland, Michigan, New York, and Ohio) reported 10–19 deaths, and 16 (Alabama, California, Colorado, Connecticut, Georgia, Louisiana, Maine, Massachusetts, Minnesota, New Jersey, North Carolina, Pennsylvania, Tennessee, Virginia, Washington, and Wisconsin) reported one–nine deaths. ^††^ Overdose deaths that occurred during January–June 2024 are preliminary and incomplete because not all deaths that occurred during this time frame were entered at the time of analysis; these data are required to be reported into SUDORS by January 24, 2025. Preliminary data were downloaded from SUDORS on November 26, 2024, and represent approximately 85% of expected overdose deaths.

Carfentanil was detected in 513 overdose deaths during January 2021–June 2024 (Figure 3). The number of overdose deaths with carfentanil detected was low during January 2021–June 2023 (≤30 per 6-month period). However, deaths with carfentanil detected increased 503.4% from 29 during January–June 2023 to 175 during July–December 2023, and increased at least another 36.0% to at least 238 during January–June 2024,^§§§§^ representing a total increase of 720.7% from the first half of 2023 to the first half of 2024. The average number of deaths with carfentanil detected sharply increased from 3.3 per month during January 2021–June 2023 to 34.4 per month during July 2023–June 2024. Among deaths with carfentanil detected during July 2023–June 2024, 86.9% had IMF co-detected. During January 2023–June 2024, carfentanil was detected in at least one overdose death in 37 states and at least 20 deaths in eight states, all east of the Mississippi River (Figure 3).

## Discussion

Although both the number of drug overdose deaths overall and the number with IMFs detected began to decline across the United States in late 2023, overdose deaths remain high. Percentages of overdose deaths with IMFs detected were stable (approximately 70%–80%) during 2021–2024, except in the West, where percentages increased from 48.5% to 66.5%. Despite declines, recent sharp increases in overdose deaths with carfentanil detected, although rare, highlight the ever-changing illegal drug supply and threaten progress in reducing overdose deaths.

In the Northeast, Midwest, and South regions, the percentages of overdose deaths with IMFs detected was high (approximately 70%–80%) and stable from 2021 to June 2024, suggesting that IMFs have saturated the illegal drug supply (i.e., IMFs have become the dominant illegal opioid and have stabilized at a high level in the supply). Over the previous decade, the proliferation of potent rapid-acting IMFs that substantially increased fatal overdose risk and resulted in sharp rises in overdose deaths might have outpaced the impact of large-scale national, state, and local efforts to reduce drug overdoses (*1*–*3*). However, saturation of the drug supply with IMFs resulting in a more stable supply might now lead to lower overdose risk as persons using drugs might have increased tolerance^¶¶¶¶^ and might also be more aware that products contain IMFs. In addition, recent mixing of non-opioid drugs (e.g., xylazine) into the fentanyl supply might reduce fentanyl purity, thereby potentially decreasing overdose risk***** (*7*). In this context of suggested saturation of the drug supply with IMFs and the potential for reduced overdose risk, more persons might be able to avoid or survive overdose and subsequently benefit from overdose prevention programs that have been implemented; this could be partially responsible for the decline in overdose deaths starting in late 2023. Continued and expanded implementation of these programs, including naloxone distribution and increasing access to treatments for substance use disorders,^†††††^ might result in sustained and continued declines in drug overdose deaths. However, the potential for increases in overdoses remains if drugs more potent than fentanyl, including carfentanil, continue to adulterate the supply.

IMFs entered western U.S. drug markets later than other regions, likely because of challenges mixing fentanyl into the black tar heroin that was more common in the West (*1*). Recent increases in counterfeit pills containing IMFs, particularly in the West, have helped IMFs infiltrate the market, increasing overdose risk (*8*). Consistent with more recent proliferation into western markets, the West experienced recent large increases in the number and percentage of deaths with IMFs detected, approaching 70% in early 2024. If the West is similar to other regions where deaths plateaued when ≥70% of overdose deaths had IMFs detected, this region might soon experience a lasting plateau or decrease in overdose deaths as the drug supply approaches potential saturation with IMFs.

Although still rare, overdose deaths with carfentanil detected increased approximately sevenfold starting in mid-2023. Because carfentanil is 100 times more potent than fentanyl (*4*), overdose deaths could substantially increase if carfentanil further infiltrates the drug supply, as evidenced by previous outbreaks (*5*,*6*). The geographic spread (37 states) and substantial codetection with IMF (87%) are markedly different from what was observed during the emergence of carfentanil in overdose deaths during 2016–2017, in which outbreaks were localized, and <25% of deaths had fentanyl co-detected (*5*,*6*). The potential mixing of carfentanil into fentanyl products as an adulterant raises concern that its presence might be unknown to persons using drugs, reminiscent of the way that fentanyl was first introduced as an adulterant of heroin (*1*). Rigorous monitoring of carfentanil and other opioids more potent than fentanyl, such as some nitazene analogs that are currently rare but persistent in overdose deaths,^§§§§§^ is warranted because of increased fatal overdose risk that could threaten recent progress in reducing overdose deaths.

To sustain reductions in overdose deaths, implementation of prevention efforts focused on the risks of IMFs, including carfentanil, is critical. These efforts include education about the risks of substance use and dangers of using pills that are not prescribed because they could be counterfeit and contain IMFs; drug checking services to help persons know what their drugs contain; increased access to naloxone for persons who use drugs and other laypersons, to ensure timely administration to reverse opioid overdoses; and messaging about additional risk reduction approaches (e.g., not using drugs while alone).^¶¶¶¶¶^ In addition, fentanyl test strips can help persons who use drugs identify products that contain fentanyl but cannot distinguish carfentanil from fentanyl (*9*). Because carfentanil is a fentanyl analog, prevention efforts focused on IMFs overall will also be effective for reducing overdoses specifically involving carfentanil. However, given carfentanil’s high potency, more doses of naloxone and faster overdose response might be required to prevent death (*10*). Combining these risk reduction efforts with efforts to reduce drug use, both through preventing drug use initiation and increasing access to treatments for substance use disorders (e.g., medications for opioid use disorder),****** could lead to lasting decreases in overdose deaths that could withstand changes in the drug supply.

### Limitations

The findings in this report are subject to at least three limitations. First, depending on the analysis, 46 or 50 jurisdictions were included; thus, results might not be generalizable to the entire United States. Second, postmortem toxicology testing lacks standardization across and within jurisdictions and might result in differential carfentanil detection. Finally, 2024 data are preliminary; not all overdose deaths that occurred have been reported to SUDORS yet. These limitations likely underestimate overdose deaths with carfentanil detected. Therefore, the increases in deaths with carfentanil detected are likely larger than those presented in this report.

### Implications for Public Health Practice

Reductions in unintentional and undetermined intent drug overdose deaths overall and with IMFs detected described in this report coincide with provisional estimates showing projected decreases in overdose deaths nationally in 2023 and into 2024.^ ††††††^ Importantly, most overdose deaths in the United States still have IMFs detected. In the West, deaths increased into 2023 as the percentage with IMFs detected approached levels similar to those seen in the rest of the country. Efforts focused on preventing deaths involving IMFs, including carfentanil and other analogs, such as maintaining and improving distribution of risk reduction tools, increasing access to and retention in treatment for substance use disorders, and preventing drug use initiation, might result in sustained decreases in overdose deaths. Finally, educational and response efforts that can rapidly adapt to the potential for increased distribution of drugs more potent than fentanyl, such as carfentanil, are needed and might avert or mitigate new increases in overdose deaths.

## Footnotes

* https://www.cdc.gov/nchs/nvss/vsrr/prov-drug-involved-mortality.htm (Accessed September 13, 2024).

†
https://www.cdc.gov/overdose-prevention/data-research/facts-stats/sudors-dashboard-fatal-overdose-data.html

§ https://www.cdc.gov/nchs/nvss/vsrr/drug-overdose-data.htm (Accessed September 13, 2024).

¶ https://www.health.ny.gov/diseases/aids/consumers/prevention/oduh/docs/2024-06-07_health_alert.pdf; https://www.ohioattorneygeneral.gov/Media/News-Releases/September-2023/Warning-Issued-for-Reemerging-Synthetic-Opioid-in; https://www.millenniumhealth.com/signalsalert/carfentanil2024/

**
https://www.cdc.gov/overdose-prevention/data-research/facts-stats/about-sudors.html

††
https://www.cdc.gov/overdose-prevention/php/od2a/state.html

§§ Fentanyl was classified as likely illegally manufactured using toxicology, scene, and witness evidence. For the 9.9% of deaths with fentanyl detected that had insufficient evidence for classification as illegal or prescription, fentanyl was classified as illegal because the majority of fentanyl overdose deaths involve illegal fentanyl. All fentanyl analogs except pharmaceutical analogs (i.e., alfentanil, remifentanil, and sufentanil) were included as IMFs. Among deaths with IMFs detected, 98.4% had IMFs listed as a cause of death.

¶¶ U.S. Census Bureau regions were used to stratify jurisdictions into geographic regions (https://www2.census.gov/geo/pdfs/maps-data/maps/reference/us_regdiv.pdf). Region analysis included nine of nine jurisdictions in the Northeast Region, 10 of 12 jurisdictions in the Midwest Region, 16 of 17 jurisdictions in the South Region, and 11 of 13 jurisdictions in the West Region.

*** Overdose deaths during January–June 2024 are required to be entered into SUDORS by January 24, 2025; thus, data are incomplete because not all deaths that occurred during this time frame were entered at the time of analysis. These data have not undergone extensive quality-control checks implemented after the reporting deadline. However, two independent analysts conducted appropriate data quality checks on IMFs and carfentanil. Preliminary data were downloaded from SUDORS on November 26, 2024.

††† Because not all overdose deaths that occurred during January–June 2024 have been entered into SUDORS, numbers of deaths overall and with IMFs detected during this time frame are underestimated and not presented. Percentages with IMFs detected during this time frame are restricted to deaths with toxicology information entered; these are expected to be accurate estimates of the percentages of all overdose deaths with IMFs detected.

§§§ For analyses of carfentanil co-detected with IMF, IMF includes illegally manufactured fentanyl, but does not include illegally manufactured fentanyl analogs. Carfentanil itself is an illegally manufactured fentanyl analog.

¶¶¶ Alabama, Alaska, Arizona, Arkansas, Colorado, Connecticut, Delaware, District of Columbia, Florida, Georgia, Hawaii, Idaho, Illinois, Indiana, Iowa, Kansas, Kentucky, Louisiana, Maine, Maryland, Massachusetts, Michigan, Minnesota, Mississippi, Missouri, Montana, Nebraska, Nevada, New Hampshire, New Jersey, New Mexico, New York, North Carolina, Ohio, Oklahoma, Oregon, Pennsylvania, Rhode Island, South Carolina, South Dakota, Tennessee, Utah, Vermont, Virginia, Washington, and West Virginia. For inclusion, jurisdictions were required to report ≥75% of deaths in their jurisdiction in each 6-month period during January 2021–December 2023.

**** Jurisdictions were included if they reported any drug overdose deaths to SUDORS during January 2023–June 2024. North Dakota was not included because it is not funded for SUDORS. For January–December 2023, all jurisdictions except one reported ≥90% of drug overdose deaths in their jurisdiction. For January–June 2024, the percentage of overdose deaths reported by jurisdiction was not calculated because data are preliminary and incomplete. Texas and Wyoming only include data on deaths during January–June 2024 per funding agreement.

†††† 45 C.F.R. part 46.102(l)(2), 21 C.F.R. part 56; 42 U.S.C. Sect. 241(d); 5 U.S.C. Sect. 552a; 44 U.S.C. Sect. 3501 et seq.

§§§§ The increase in the number of deaths with carfentanil detected from July–December 2023 to January–June 2024 is at least 36.0%. The actual increase is likely larger because not all overdose deaths that occurred during January–June 2024 had been entered in SUDORS at the time of publication.

¶¶¶¶
https://nida.nih.gov/publications/drugfacts/understanding-drug-use-addiction

*****
https://www.cfsre.org/images/content/reports/drug_checking/Fentanyl_Purity_Potency_and_Synthesis_August_2022.pdf

††††† https://www.hhs.gov/overdose-prevention/; https://www.cdc.gov/overdose-prevention/php/od2a/about.html; https://aspe.hhs.gov/sites/default/files/documents/facbce1704035fded1034192d148304d/buprenorphine-practice-guideline-early-impacts.pdf; https://www.cdc.gov/overdose-prevention/media/pdfs/OD2A-Case-Studies-State-and-Local-Integration-508.pdf

§§§§§ https://www.cfsre.org/nps-discovery/trend-reports/nps-opioids/report/49?trend_type_id = 2; https://www.cdc.gov/overdose-prevention/data-research/facts-stats/sudors-dashboard-fatal-overdose-data.html

¶¶¶¶¶
https://www.cdc.gov/overdose-prevention/prevention/index.html
